# A study on the relationship between yoga exercise intervention and the comprehensive well-being of female college students

**DOI:** 10.3389/fpsyg.2024.1425359

**Published:** 2024-07-08

**Authors:** Lanjuan Liu, Dandan Liu, Cheng Liu, Yanran Si

**Affiliations:** ^1^School of Physical Education, Shanghai Normal University, Shanghai, China; ^2^Department of Fitness Instruction, Zhengzhou Physical Education Vocational College, Henan, China; ^3^Department of Physical Education, Donghua University, Shanghai, China; ^4^Department of Physical Education, Shanghai International Studies University, Shanghai, China

**Keywords:** yoga, exercise intervention, comprehensive well-being, female college students, relationship

## Abstract

**Background:**

Due to the influence of theories, tools, and methodologies in studying well-being, sports science has predominantly focused on subjective well-being, with less attention given to psychological well-being and even less to the integrated study of comprehensive well-being. This study aims to analyze the relationship between yoga exercise intervention and the comprehensive well-being of college students and to explore the mechanism of a yoga exercise intervention to improve the comprehensive well-being of female college students.

**Methods:**

With 92 female college students as subjects, the “Comprehensive Well-being Scale” was used, and research methods such as yoga exercise intervention, questionnaire surveys, qualitative analysis, expert interviews, and statistical analysis were employed to investigate the role of a yoga exercise intervention on the comprehensive well-being of female college students.

**Results:**

Among the nine dimensions of comprehensive well-being, the three dimensions of subjective well-being and the two dimensions of psychological well-being (health concern and personality growth) of female college students were significantly improved. Additionally, four other dimensions of psychological well-being also showed significant improvement. Furthermore, the improvement in the life satisfaction of female college students’ subjective well-being was mainly achieved through yoga meditation, while partner yoga posture practice could help individuals gradually form a stable pattern of altruistic behavior.

**Conclusion:**

Yoga exercise intervention can improve the comprehensive well-being of female college students and can be an effective counseling method for college students’ mental health education.

## 1 Introduction

Well-being refers to an individual’s holistic evaluation of their life quality based on their standards, encompassing a psychological state of overall satisfaction with life and a comprehensive indicator reflecting their quality of life (Chu et al., 2023). Well-being can be divided into subjective and psychological well-being; despite numerous differences, they are interrelated (Keyes et al., 2002). Research on the relationship between physical exercise and well-being began in the 1960s, employing qualitative, quantitative, or mixed methods. Studies have explored this relationship from various perspectives, including the emotional and cognitive dimensions of subjective well-being, the mechanisms of influence, factors affecting psychological well-being and their associations, involving participants ranging from adolescents to the elderly (Ryff, 1989; Ferguson et al., 2012; Donizzetti, 2023).

Research utilizing data from the UK’s International Society of Sport Psychology (ISSP), the US’s Behavioral Risk Factor Surveillance System (BRFSS), Germany’s computer-assisted personal interview (CAPI), as well as Baptist Health South Florida (BHSF) and Environment, Biodiversity & Soil Security (EBSS) data, has found that individuals who participate in physical activities report higher levels of personal well-being, with this effect being particularly pronounced among male participants. The primary mechanisms identified include the “health mechanism, “the “social capital mechanism, “and the “public service mechanism “(Sun et al., 2022).

However, focused research on the impact of specific sports interventions on participants’ well-being and systematic studies addressing gender differences in research participants have been relatively scarce. Furthermore, the orientations toward subjective and psychological well-being show a trend toward integration, necessitating a combined approach to understanding well-being comprehensively (Botha et al., 2019). Nevertheless, due to the influence of theories, tools, and methodologies in studying well-being, sports science has predominantly focused on subjective well-being, with less attention given to psychological well-being and even less to the integrated study of comprehensive well-being.

Despite some research focusing on female college students, previous studies have failed to adequately consider the impact of gender differences on perceptions of overall well-being due to limitations inherent in traditional research paradigms. Prior research has indicated that, compared to their male counterparts, female college students are more susceptible to stress and mental health issues, which significantly affect their overall well-being (Barbayannis et al., 2022). For instance, academic pressures, societal expectations, and personal health concerns often result in higher incidence rates of stress-related illnesses among female college students (Howard et al., 2006). The physiological and psychological stressors faced by female students differ from those encountered by males and include, but are not limited to, heightened academic pressure due to gender-specific societal expectations, complex social identity issues influenced by cultural and interpersonal factors, and unique health concerns such as menstrual and reproductive health, as well as higher prevalence rates of anxiety and depression (Hall et al., 2006). Since yoga integrates physical postures, meditation, and breathing exercises, it offers unique benefits in promoting relaxation, reducing anxiety, improving overall mental health, and alleviating gender-specific stressors (Lim and Hyun, 2021). Consequently, women are more likely to prioritize engaging in activities closely related to psychological and emotional health, making them ideal subjects for studying the effects of yoga interventions.

Based on this premise, this study focuses on female college students, seeking to identify effective yoga intervention strategies tailored for women. This approach addresses a critical gap in the existing literature and contributes to developing gender-sensitive health promotion programs, ultimately enhancing female college students’ overall quality of life. Additionally, it is crucial for supporting female students’ physical and mental health development and assisting policymakers and educational institutions in designing rational and healthy plans. Therefore, this study aims to explore the effects of yoga interventions on female college students’ physiological and psychological aspects, using a combination of yoga exercise interventions, surveys, qualitative analysis, expert interviews, and statistical analysis. It delves into the process and mechanisms by which yoga improves various aspects of comprehensive well-being among female college students to reveal the relationship between yoga interventions and their comprehensive well-being fully, enhance college sports by optimizing intervention strategies, improve psychological health and overall well-being of college students and thereby contribute to the implementation of the Healthy China strategy and the development and improvement of related policies.

## 2 Research subjects and methods

### 2.1 Research subjects

One hundred sophomore female students from a normal university in Shanghai were selected for a yoga therapy teaching intervention using convenience sampling. Inclusion criteria were: (1) informed consent and voluntary participation; (2) no other psychological interventions received in the past 3 months, no history of surgery, and no contraindications to yoga exercise. Exclusion criteria were: (1) recent participation in related psychological interventions; (2) existing health problems such as heart disease or protrusion of intervertebral discs. Drop-out criterion: poor compliance after inclusion, with fewer than two exercise sessions attended. Adhering to the principles of voluntariness and confidentiality, a random grouping method was used to divide the 100 students into two groups, resulting in 92 participants for the effective intervention, with 46 in the control group and 46 in the yoga group, averaging 19 ± 1.15 years of age. There was no statistically significant difference in age, major or other general information between the two groups (*p* > 0.05), making them comparable (Table 1).

**Table 1:** Demographics of students (*N* = 92).

Terms	Yoga group		Control group	
	*n* = 46	%	*n* = 46	%
Gender				
Female	46	100.0	46	100.0
Age				
18 ~ 19	41	89.1	9	19.6
20 ~ 21	5	10.9	37	80.4
Major				
Education	14	30.4	15	32.6
Literature	6	13.0	9	19.6
Management	11	23.9	8	17.4
Economics	3	6.5	4	8.7
Engineering	5	10.9	5	10.9
Science	5	10.9	2	4.3
Law	2	4.3	3	6.5
Home location				
City	21	45.7	24	52.2
Township	25	54.3	22	47.8

### 2.2 Literature review and expert interview

This study reviews and synthesizes the existing literature to systematically understand the latest research trends in the relevant fields, both domestically and internationally. The literature review includes a detailed analysis of various well-being assessment tools and their measurement dimensions. Notably, we reference the study by Linton et al. (2016), who examined 99 self-report scales used to assess adult well-being, providing a broader perspective on the different dimensions of well-being, such as subjective well-being, psychological well-being, and social well-being (Linton et al., 2016). Linton identified key themes in well-being assessment tools, including emotional well-being, mental health, social functioning, and life satisfaction. By describing these tools and their reflected themes, we aim to understand the multidimensional nature of well-being comprehensively. This understanding helps to justify better the choice of well-being measurement tools in our study, ensuring that it captures all relevant aspects of comprehensive well-being in the target population.

In well-being research, subjective well-being typically includes emotional experiences and life satisfaction (Maddux, 2017). Emotional experiences refer to the positive or negative emotional states people experience in their daily lives, while life satisfaction refers to an individual’s perception and evaluation of their overall quality of life (Maddux, 2017). Measuring these dimensions is crucial for a comprehensive understanding of individual well-being. Additionally, psychological well-being involves aspects such as personal growth, self-acceptance, and life goals, reflecting an individual’s evaluation of their development (Maddux, 2017). Social well-being focuses on interpersonal relationships and social support, emphasizing an individual’s position within social networks and the level of support received (Maddux, 2017). Based on Linton et al.’s study, we further analyzed the performance of these well-being dimensions in different cultural and social contexts. For instance, Western cultures emphasize individual achievement and independence, potentially placing more importance on psychological well-being, while Eastern cultures emphasize collectivism and social harmony, possibly focusing more on social well-being. These cultural differences should be carefully considered when selecting and applying well-being measurement tools to ensure validity and reliability.

Moreover, we explored the applicability of well-being measurement tools among different populations, including adolescents, adults, and the elderly, as there may be significant differences in well-being experiences and expressions across different age groups. Therefore, adaptive measurement tools need to be employed in research design. By thoroughly analyzing the advantages and limitations of different measurement tools and their applicable ranges, we aim to select tools that accurately reflect the well-being levels of the study participants.

Based on the literature review, we conducted in-depth interviews with experts and scholars in the field. The feedback from these experts on our preliminary experimental intervention plan helped us refine the research methods, ensuring that the chosen well-being measurement tools are robust, reliable, and relevant to the study objectives. We identified current hotspots and challenges in well-being research through expert interviews, such as capturing changes in dynamic environments, addressing well-being differences across cultural backgrounds, and effectively enhancing individual well-being levels through intervention programs. This finding provided valuable research insights and methods, enabling us to consider more variables and influencing factors in the experimental design, aligning our research methods with best practices in the current well-being research field and effectively distilling the complex multidimensional nature of well-being.

Based on the literature review and expert interviews, we finalized the experimental intervention plan. The revised plan includes more detailed and refined research methods for measuring well-being and fully incorporates the latest research trends and expert recommendations.

### 2.3 Intervention scheme

The subjects were divided into two groups: a yoga group and a control group. Yoga exercise interventions were implemented for the students in the experimental group for 10 weeks. No physical exercise intervention is arranged for the control group students. The specific experimental intervention scheme is as follows:

#### 2.3.1 Composition of the yoga intervention group

The group consisted of one tutor with a master’s degree (a professional yoga teacher with 13 years of experience in university-level yoga instruction), one master candidate, and three monitors (undergraduate students). The primary rationale for selecting a 10-week duration for the exercise intervention is threefold: Firstly, previous studies have demonstrated that a 10-week period is sufficient to observe significant physical and psychological changes associated with regular yoga practice (Schoch-Ruppen et al., 2018). Secondly, the 10-week duration aligns with the academic semester schedule, minimizing disruption to participants’ normal daily routines. Lastly, a 10-week period allows participants to establish a consistent yoga practice, which is crucial for achieving significant improvements in well-being (Polsgrove et al., 2016).

#### 2.3.2 Pre-experiment

A complete experiment for 60 female students was organized before the official experiment to refine the intervention content after addressing issues identified during the pre-experiment and finalizing the content of the intervention course (Table 2).

**Table 2:** Content of the yoga intervention.

Week	Practice content
1	Posture Theme: Establishing Foundation. Class Requirement: Observe breathing and attempt to focus on the breath. Meditation Theme: Observing Breath (10 min); Seated Breathing Exercise (10 min); Posture Practice: Mountain Pose - Prayer Pose - Standing Forward Bend - Plank Pose - Child’s Pose - Cobra Pose - Downward Dog - Cat Stretch - Happy Baby Pose (60 min); Relaxation Technique: Corpse Pose (10 min); Posture Theme: Dynamic Foundation. Class Requirement: Feel the breath in dynamic postures. Meditation Practice: Focused Breathing (10 min); Seated Breathing Exercise: Natural Breath + Abdominal Breathing (10 min).
2	Posture Practice: Seated Cat-Cow Pose (Dynamic) - Single Leg Seated Forward Bend - Seated Spinal Twist (Both Sides)- Child’s Pose – Both sides of Back Stretch (Dynamic) - Cat-Cow Pose (Dynamic) - Tiger Pose (Dynamic) - Downward Dog - Warrior II - Reverse Warrior (Dynamic) - Extended Side Angle Pose (Both Sides) - Child’s Pose - Locust Pose (Dynamic) – Half-bow Pose - Bridge Pose (Dynamic) - Reclining Bound Angle Pose (60 min) Relaxation Technique: Corpse Pose (10 min)
3	Posture Theme: Foundation consolidation. Classroom requirements: Deep and prolonged breaths to release stiffness and tension, consciously relax the body, and reconcile with pain sensations. Meditation Theme: coexisting with emotions (10 min) Sitting meditation and breath regulation (Natural Breathing + Abdominal Breathing) (10 min) Posture Practice: Seated Cat-Cow Pose -Side Stretch - Seated Spinal Twist (Both Sides) Single Leg Seated Forward Bend (Both Sides) - Cat-Cow Pose - Tiger Pose (Dynamic) - Downward Dog - Warrior II -Reverse Warrior (Dynamic) - Extended Side Angle Pose (Left) - Downward Dog - Child’s Pose - Downward Dog - Warrior II - Reverse Warrior - Extended Side Angle Pose (Right) - Downward Dog - Extended triangle pose (left) - Downward Dog - Extended triangle pose (right) - Downward Dog - Standing Forward Bend - Mountain Pose -Plank pose - Child’s Pose - Cobra Pose - Locust Pose (Dynamic) - Bridge Pose (Dynamic) - Happy Baby Pose - Reclining Bound Angle Pose (60 min) Relaxation Technique: Corpse Pose (10 min)
4	Meditation Theme: Positive Mindset Asana Theme: Foundation Consolidation Class Requirements: Throughout the practice, awareness follows the breath and the breath relaxes the body. Asana practice is the same as week 3
5	Meditation Theme: Love Yourself Asana Theme: Whole Body Stretching Class Requirements: Breathe deeply and slowly, treat yourself slowly and gently during the practice. Supine Meditation (Abdominal Breathing) 20 min One-legged Sit & Stand - Sit & Stand - Vajra Sit - Side Lumbar Stretch - Reverse Prayer Pose - Half Camel Pose - Big Bow Pose - Cobra Pose -Bow Pose - Happy Baby Pose - Fish Pose - Supine Bound Angle Pose - Stall Body Pose Resting Technique
6	Class Requirements: Experience the increase in breathing space when the chest cavity expands, improving breathing quality. Meditation Theme: Stress Relief (10 min) Pose Theme: Opening the Heart (10 min) Supine Meditation (Abdominal Breathing) (10 min) Pose Practice:Seated Side Stretch - Seated Cow Face Pose - Bound Angle Pose - Revolved Knee-to-Finger Pose - Diamond Pose - Cat-Cow Pose - Extended Cat Stretch-Sphinx Pose - Cobra Twist - Downward Dog - Crescent Pose - Backbend Crescent Pose - Grand Salutation - Downward Dog - Warrior I - Warrior I Backbend - Half Pigeon Pose (left and right) - Locust Pose - Bow Pose - Fish Pose - Supine Single-Leg Twist (left and right) - Happy Baby Pose - Supine Bound Angle Pose (60 min) Rest Technique: Corpse Pose Rest (10 min)
7	Class Requirement: Maintain steady breath, feel the body exerting effort, and maintain stability in mind and muscles. Posture Theme: Stabilizing the Body Meditation Theme: Enhancing Mental Energy (10 min) Back-to-Back Seated Meditation (Abdominal Breathing + Natural Breathing) (10 min) Posture Practice: Seated Side Stretch - Partner Seated Spinal Twist - Assisted Seated Forward Bend - Thunderbolt Pose - Cow Face Pose - Cat-Cow Pose - Tiger Balance - Tiger Bow - Child’s Pose - Gate-Latch Pose (Both Sides) - Downward Dog - Crescent Lunge - Half Monkey Pose - Downward Dog - Crescent Lunge - Half Monkey Pose - Plank Pose - Child’s Pose - Cobra Pose - Flying Fish Pose - Bow Pose - Bridge Pose - Boat Pose - Reverse Plank - Reclining Bound Angle Pose - Happy Baby Pose (60 min) Relaxation Technique: Corpse Pose (10 min)
8	Meditation Theme: Rejuvenation Asana Theme: Backbends & Stretches Class Requirements: Feel the flexibility of the spine in the body, the opening of the chest and the stretching of the legs. Back to Back Sitting Meditation (Abdominal Breathing) 15 min Sitting Standing Side Waist Stretch - Double Spinal Twist - Assisted Sitting Standing Forward Bend - Vajra Sit - Cow Face Pose - Cat Cow Pose - Tiger Balance -Tiger Bow Pose (right and left) -Da Bai Pose -Door Latch Pose (right and left) -Downward Dog Pose -Crescent Moon Pose (left) -Demi-God Monkey Pose (left) -Downward Dog Pose Crescent Pose (right) - Semi-God Monkey Pose (right) - Big Worship Pose + Downward Facing Dog Pose - Double Boat Pose - Phantom Chair Pose + Hip Bridge Pose - Supine Bound Angle Pose - Fish Play Pose Resting Technique
9	Meditation Theme: Learning Gratitude Asana Theme: Focus on your partner Class Requirements: In asana, pay attention to and protect your partner, focus on the feeling of feedback from both bodies, and give each other stable support. Sit-to-stand meditation (alternating left and right nostril breathing technique) 15 min Seated Side Lumbar Stretch - Spinal Twist for Two - Assisted Seated Forward Bend - Cat Cow Pose - Tiger Bow Pose - Big Cat Stretch - Big Bye Pose + Camel Pose -Big Worship Pose + Downward Facing Dog Pose -Pair Phantom Chair Pose -Pair Tree Pose -King Bird Pose -Standing Forward Bend -Crescent Moon Pose -War I -Battle III - Plank Pose - Big Bye Pose - Cobra Pose + Phantom Chair Pose - Cat Stretch Pose - Happy Baby Pose - Stall Body Rest Pose
10	Class Requirement: In partner yoga, feel the same sensations as your partner and strive to assist your partner in completing the movements. Posture Theme: Assisting Partner Meditation Theme: Cultivating Empathy (10 min) Breathing Exercise: Abdominal Breathing from Standing Mountain Pose (10 min) Posture Practice: Seated Side Stretch - Partner Seated Spinal Twist - Assisted Seated Forward Bend - Partner Tiger Pose Balance - Child’s Pose + Camel Pose - Child’s Pose + Downward Dog - Cobra Pose + Chair Pose - Partner Tree Pose - Partner Warrior III - Partner Boat Pose - Chair Pose + Bridge Pose (60 min) Relaxation Technique: Corpse Pose (10 min)

#### 2.3.3 Form of intervention

Led by the teacher, the students in the yoga group underwent thematic yoga courses aimed at enhancing comprehensive well-being, including 10 min of yoga meditation, 10 min of breathing exercises, 60 min of moderate-intensity postural exercises, and 10 min of yoga relaxation, once per week (Table 2). The students in the control group received no yoga exercises. The tutor was responsible for full-time supervision and instruction during the teaching period. The graduate student assisted in implementing the 10-week yoga teaching, interacting with students during and after class to collect their exercise feedback. Three class monitors selected from the students were responsible for ensuring their classmates’ timely participation in the exercises.

#### 2.3.4 Content of the intervention course

The yoga exercise intervention content was divided into four parts: postures, meditation, breathing, and relaxation techniques, covering 10 themes: “Observing Breath & Establishing Foundation,” “Focusing on Breath & Dynamic Basics,” “Coexisting with Emotions & Foundation Strengthening,” “Positive Mindset & Foundation Strengthening,” “Self-Love & Full Body Stretch,” “Stress Relief & Open Heart,” “Enhancing Psychological Energy & Stabilizing Body “Revitalizing & Backbends and Stretching,” “Learning Gratitude & Paying Attention to Peers,” and “Cultivating Empathy & Assisting Peers,” with one theme per week for teaching interventions.

#### 2.3.5 Rationale for selecting the above intervention content based on literature review, expert interviews, and pre-experimental refinement

(1) Comprehensive Enhancement of Multidimensional Well-Being. Asanas (Postures): Physical postures and movements directly improve physical health and enhance physical fitness, indirectly boosting psychological health and emotional management capabilities. Meditation: Meditation helps increase concentration, reduce stress and anxiety, and improve emotional stability and psychological resilience. Pranayama (Breathing Exercises): Breathing exercises help regulate the autonomic nervous system, enhancing overall mental and physical health. Relaxation Techniques: Systematic relaxation practices help eliminate physical and mental tension, enhancing overall well-being. (2) Scientific Basis for Thematic Design: Each weekly theme is based on established yoga and psychology research findings. For example, the theme “Observing Breath & Establishing Foundation” aims to help participants build a basic understanding of yoga and physical foundation through initial breathing exercises and basic postures, aligning with beginners’ needs and learning curves. Themes such as “Stress Relief & Heart Opening” and “Boosting Mental Energy & Stabilizing the Body” directly correspond to specific mental health benefits achievable through yoga practice, which are well-supported by previous academic literature. (3) Gradual Progression of Practice: The course content is arranged from basic to advanced, gradually increasing in difficulty and complexity. This design aligns with the principle of progression in educational theory, helping participants gradually adapt to and embrace yoga practice while progressively experiencing yoga’s physical and mental benefits. (4) Practicality and Feasibility: The time allocation and content design of each part (e.g., 10 min of meditation and 60 min of asana practice) consider students’ daily life rhythms and study schedules, making the intervention plan practical, feasible, and sustainable.

#### 2.3.6 Additional research design

This study adopts a convergent design within a mixed-methods approach. Quantitative data will be collected using a comprehensive well-being scale before and after the experiment. Qualitative data will be collected through semi-structured interviews and meditation diaries during and after the experiment. The results of both data types will be compared and integrated to provide a comprehensive understanding of the research question. This approach allows for the validation and enrichment of research findings, leading to a more robust interpretation of the impact of yoga intervention on students’ well-being.

#### 2.3.7 Quality control of the experiment

The following quality control measures were taken to ensure the effective completion of the intervention scheme by students. A WeChat group was established to encourage students to upload practice photos, facilitating the exchange of practice experiences among students and between students and teachers. In addition, teachers were available for immediate question-and-answer sessions, enhancing students’ interest in participation. Finally, attendance and experience sharing were included in the assessment scores for this practice course, with incentive measures adopted to improve student compliance.

### 2.4 Survey method

To better address issues of cultural adaptability, reliability, and standardization of measurement tools, this study adopted the Multiple Happiness Questionnaire (MHQ). The MHQ, developed by Professor Yuanjiang Miao in 2003, integrates theories and measurement indicators of subjective and psychological well-being (Zhu, 2022). It has been widely used in subsequent research to evaluate the comprehensive well-being of college students and middle-aged to elderly fitness groups. The MHQ has a Cronbach’s alpha coefficient ranging from 0.700 to 0.919, a McDonald’s omegas coefficient ranging from 0.35 to 0.95, and a split-half reliability coefficient between 0.635 and 0.820, demonstrating good reliability and validity (Wei, 2011; Cramer et al., 2013; Jiapeng and Chunyu, 2021).

The primary reasons for choosing to use the Mental Health Questionnaire (MHQ) in this study are as follows: The MHQ is specifically designed to capture subjective and psychological well-being, which are highly aligned with our research objectives. The comprehensive nature of the MHQ encompasses nine dimensions of well-being, including life satisfaction, positive and negative emotions (subjective well-being), vitality, health concerns, self-worth, altruistic behavior, friendly relationships, and personal growth (psychological well-being). This comprehensive nature ensures a thorough evaluation of the impact of yoga intervention on various aspects of well-being. Although other tools, such as the 3rd generation Interpersonal, Community, Occupational, Physical, Psychological, and Economic well-being (ICOPPE) and the MQLI (Multicultural Quality of Life Index), are also comprehensive, we chose the MHQ because their reliability and validity have been well-established among Chinese university students, our specific population of interest. Additionally, the widespread use of the MHQ in similar research contexts provides a solid foundation for comparative analysis, enhancing the validity of our study results.

Therefore, the comprehensive well-being scale chosen for this study was suitable for assessing and evaluating the impact of yoga interventions on the comprehensive well-being of female college students. The MHQ consists of 50 items, including subjective and psychological well-being aspects. Subjective well-being is divided into three dimensions (life satisfaction, positive affect, and negative affect), and psychological well-being is divided into six dimensions (Life vitality, health concern, self-worth, altruistic behavior, friendly relationships, and personality growth), totaling nine dimensions. The scale uses a 7-point scoring method, with reverse scoring for negative affect, where higher scores indicate higher levels of comprehensive well-being. Tests using a comprehensive well-being scale were conducted 1 week before and after the intervention.

### 2.5 Qualitative analysis

After the exercise intervention, semi-structured interviews were conducted in small groups based on the number of attendees to explore their genuine feelings about the yoga practice, with participant consent obtained for onsite recording and note-taking. The same person conducted interviews, each lasting about 1 h, focusing primarily on the nine dimensions of comprehensive well-being, with particular attention to students’ descriptions of their experiences. Recordings were transcribed into text and imported into Nvivo12 software for qualitative analysis. The main steps included repeatedly reading interview records, identifying initial themes and coding, and refining primary themes. Two researchers completed the coding analysis; the coding consistency percentage was 84%. Discrepancies in coding are discussed jointly to decide and achieve the highest degree of fit between coding content and themes.

### 2.6 Statistics

Data analysis was performed using SPSS 21.0 software. Quantitative data were described using mean ± standard deviation (SD). Before performing t-tests, the normality of the data was assessed using the Shapiro–Wilk test. If the data met the normality assumption (*p* > 0.05), paired t-tests were used for group comparisons. Nonparametric tests were employed if the data did not meet the normality assumption. In addition to reporting means and standard deviations, the t statistic and effect size (Cohen’s d) were also calculated.

## 3 Results

### 3.1 Comparison of MHQ and various indicators scores before and after yoga intervention in two groups of students

Before performing t-tests, the normality of the data was assessed using the Shapiro–Wilk test. The results showed that both pre-intervention data (*W* = 0.977, *p* = 0.350) and post-intervention data (*W* = 0.961, *p* = 0.120) met the normality assumption (*p* > 0.05). Therefore, paired t-tests were used for group comparisons (Table 3).

**Table 3:** Statistics of the yoga group and control group before and after intervention.

Factor	Yoga group pre-intervention (mean ± standard deviation)	Yoga group post-intervention (mean ± standard deviation)	Control group pre-intervention (mean ± standard deviation)	Control group post-intervention (mean ± standard deviation)	Yoga group t-statistic	Yoga group Cohen’s d	Control group t-statistic	Control group Cohen’s d
Comprehensive well-being	162.16 ± 38.85	219.03 ± 43.76	161.63 ± 39.11	164.67 ± 36.11	6.591	1.374	0.387	0.081
Life satisfaction	16.06 ± 6.13	25.95 ± 6.35	15.86 ± 6.14	15.64 ± 5.43	7.600	1.585	−0.182	−0.038
Positive affect	18.86 ± 6.91	32.51 ± 7.07	18.82 ± 6.70	18.34 ± 7.43	9.365	1.953	−0.325	−0.068
Negative affect	19.27 ± 7.91	12.58 ± 5.69	19.32 ± 7.68	20.63 ± 8.33	−4.657	−0.971	0.784	0.164
Life vitality	19.48 ± 6.34	27.52 ± 8.52	19.39 ± 6.51	19.26 ± 7.04	5.135	1.071	−0.092	−0.019
Health concern	16.39 ± 4.91	32.69 ± 6.83	16.34 ± 4.75	16.71 ± 5.47	13.143	2.740	0.346	0.072
Altruistic behavior	16.37 ± 6.04	21.88 ± 7.30	16.26 ± 6.06	16.58 ± 6.66	3.944	0.822	0.241	0.050
Self-worth	16.58 ± 6.22	27.98 ± 6.51	16.45 ± 6.23	16.61 ± 6.39	8.587	1.791	0.122	0.025
Friendly relationships	9.61 ± 3.07	14.04 ± 4.33	9.67 ± 3.02	9.87 ± 3.37	5.661	1.180	0.300	0.063
Personality growth	28.94 ± 12.57	56.55 ± 16.66	29.47 ± 10.94	30.01 ± 11.74	8.973	1.871	0.228	0.048

Overall Well-being: The overall well-being of the yoga group significantly improved after the intervention (mean increased from 162.16 to 219.03, *t* = 6.591, Cohen’s *d* = 1.374), indicating that yoga intervention had a significant positive impact on overall well-being. The overall well-being of the control group showed minimal change (mean increased slightly from 161.63 to 164.67, *t* = 0.387, Cohen’s *d* = 0.081), indicating no significant change (Table 3).

Specific Factors: The t-statistics and Cohen’s d values for specific factors such as life satisfaction, positive emotions, vitality, health concern, altruistic behavior, self-worth, friendly relationships, and personality growth indicated significant improvements in the yoga group. Notably, health concern (Cohen’s *d* = 2.740) and personality growth (Cohen’s *d* = 1.871) showed substantial enhancements, indicating significant improvement in these factors after the yoga intervention. Negative emotions significantly decreased in the yoga group (*t* = −4.657, Cohen’s *d* = −0.971), demonstrating that yoga intervention significantly reduced negative emotions (Table 3). In the control group, all factors were insignificant, with t-statistics and Cohen’s d values indicating no significant changes.

#### 3.1.1 Rationale for choosing this quantitative analysis

Rationale for Selection: We used t-tests to compare differences between the two groups (intervention and control) before and after the intervention. The t-test is a commonly used and effective statistical method that accurately reflects intervention effects and can handle paired and independent sample data. It is suitable for the design of this study and has high statistical power. The calculation of effect size (Cohen’s d) provides the magnitude of the intervention effect, complementing the t-test results and helping to understand the practical impact of the intervention better. Cohen’s d quantifies the actual significance of the intervention effect, providing a more intuitive explanation that aids in the application and dissemination of the results.

Alternative methods and their limitations. Descriptive statistics only: While providing basic means and standard deviations, it cannot perform group comparisons or indicate the statistical significance of the intervention. Descriptive statistics only offer a basic overview of the data, lacking quantitative evaluation of the intervention effect, making it difficult to draw strong conclusions. Other Test Methods (e.g., ANOVA): Although capable of handling multiple data groups, for this study’s two-group comparison design, the t-test is sufficient and simpler to operate, with results that are easier to interpret. ANOVA is suited for multiple-group comparisons, and its structure and interpretation are more complex, making it less suitable for this study’s two-group design.

### 3.2 Qualitative analysis of the yoga intervention group’s meditation diaries

This study further explored students’ genuine feelings and the entire change process through qualitative analysis of the exercise logs and interview content of the yoga group students. Based on initial coding, an analysis of the impact on the comprehensive well-being of female college students is conducted, divided into eight secondary themes: changes in emotional affect, physical changes, interpersonal interactions and relationships, personality growth, life satisfaction, further analyzing and summarizing tertiary nodes. Multiple checks and revisions of node names and contents and aggregation of similar nodes were performed, with examples for tertiary nodes (Table 4). The interviews did not reveal that yoga postures could enhance life satisfaction. Rather, the increase in life satisfaction was reflected in the effects of meditation, as mentioned by the participants: “I found that because my mood improved, everything around me got better, and my satisfaction with life increased”; “After meditating, I feel very satisfied and friendly toward everything around me.”

**Table 4:** Themes and coding of the post-intervention interview.

Primary nodes	Secondary nodes	Tertiary nodes	Reference points of coding	Coding examples
Effect of yoga intervention on comprehensive well-being	Perceptions of emotional and mood changes	Positive effects such as enthusiasm, pleasure, and optimism	36	Feeling attractive and full of confidence; feeling joyful inside, believing to have found one’s way of relaxation and hypnosis; experiencing great relaxation of mood;
		Alleviating negative effects like anxiety and stress	43	Anxiety and stress relieved; reduction of negative affect; encountering problems in life no longer seems so bad; body feels relaxed; I had insomnia that day, feeling restless, but after listening to light meditation music while lying in bed, I woke up the next day feeling energized; I sit cross-legged on the yoga mat, listened to light music for a few minutes, took deep breaths, and felt much better; complete relaxation of body and mind.
	Health changes	Attention and observation of one’s body	25	When I maintained a state beneficial to my body but accompanied by pain, coexisting peacefully with the pain, I told myself that this was also a healthier process; my body became more flexible; my body felt very relaxed and comfortable; my body got greatly stretched; meditation allowed me to sleep at 10 o’clock at night, curing insomnia.
	Interpersonal relationships and social environment	Caring for others, being friendly, and tolerant	27	After having a falling out with a friend, feeling extremely tormented and oppressed inside, I used simple sitting postures from yoga for abdominal breathing and meditation, reflecting on the reasons for the incident, and I found that I was no longer angry, trying to understand others, putting myself in their shoes; feeling satisfied with people and things around me inside; practicing yoga made me more open-minded and tolerant; yoga made me feel more relaxed and comfortable with classmates; I started to meet new friends, discovered and learned from their strengths, and my mindset gradually became more optimistic.
	Personality growth, introspection	Overall mood, emotional regulation, self-acceptance	42	Previously, when I encountered something, I might be overly negative, an absolute pessimist, but after learning abdominal breathing and meditation in yoga, I first calmed down when encountering something, analyzed it carefully, and did not exhaust my emotions excessively; became mentally tougher and learned how to regulate emotions; became calmer and more positive when faced with things; became more stable and gentle in dealing with people; improved my emotional defenses; felt the enhancement of my ability to control and regulated emotions
	Life satisfaction	Satisfaction with daily life, satisfaction with one’s life conditions	14	I found that the environment around me improved because of my improved mood; everything was fine, and my satisfaction with life increased. I could handle any situation with ease, feeling satisfied and friendly with everything and everyone around me inside
	Life vitality	Energy restoration, feeling physically light	28	After yoga practice, my body did not feel tired, but rather refreshed; after completing the yoga practice, I not only did not feel tired, but I was full of energy and became more relaxed
	Altruistic behavior	Mutual support, helping each other	15	In postures, I was willing to support her with all my strength, even if I was in pain; in helping her do postures beneficial to her health, I felt that our relationship had become closer; when we supported each other, there was a magical feeling, we gave each other energy, and it felt very warm; I was willing to help her as much as I can.
	Self-affirmation, self-worth recognition	Belief in one’s abilities, feeling successful, and having a sense of achievement	23	Through continuous perseverance and practice, completing previously impossible movements, breaking through my limits repeatedly, and believing that I can also do it, yoga makes me more confident. I have gained great confidence in my figure; in the process of continuous attempts, I have gained a great sense of achievement and satisfaction

The reference points and examples in the secondary nodes mainly focus on positive effects like experiencing love, happiness, pleasure, pride, optimism and alleviating negative effects. Some students mentioned that “I feel very relaxed; I became more relaxed and happy after meditating”; “It helped me become more patient, kind, happy, and fulfilled.”; “After meditating, I indeed felt much calmer, able to eliminate many adverse emotions, and face the pandemic and lockdowns calmly.” “Yoga and meditation greatly relax the mood, shedding some of the anxiety and displeasure from study and life, completely letting go, effectively reducing anxiety, restlessness, and other emotions.” Some participants mentioned: “When I maintained a state that is beneficial but painful for the body, coexisting peacefully with the pain, I told myself that this is also a process toward better health,” “My body has become more flexible,” “The body is greatly stretched,” “I found that meditation allows me to sleep at 10 o’clock at night, curing my insomnia.” These results indicate that yoga postures help the body become softer and more stretched, gradually becoming healthier through enduring pain. Psychologically, participants mentioned, “I’m more willing to open my mind and welcome everything with an open and curious attitude.” These results indicate that yoga postures help individuals stretch stiff bodies effectively, and meditation can cure insomnia.

Some participants mentioned: “After learning yoga’s abdominal breathing and meditation, I calm down first when encountering issues, analyze carefully, and do not overconsume my emotions”; “My inner self has become more resilient, and I have learned to regulate my emotions”; “I can distinctly feel my ability to control and regulate emotions has significantly strengthened.” These results indicate that meditation enabled a clear process of emotional self-acceptance, fostering self-care and love. In yoga meditation, some students described, “After each potential-unlocking yoga meditation, my thoughts become clearer, more goal-oriented, and my inner self feels full of confidence and strength.” These results indicate that persistence in action and meditation can significantly enhance self-worth within comprehensive well-being.

Several students described: “Grateful for our companionship, for the fateful encounters in our lives, thankful for being together, grateful for witnessing each other’s lives, love you all, wishing everyone well,” “Had a conflict with a friend today, felt a bit upset, but after listening to the meditation on perceiving interpersonal boundaries, felt somewhat relieved, realizing the need to face relationships more openly.” These results indicate that Yoga Meditation creates friendly and harmonious interpersonal relationships, relieves interpersonal difficulties, and enhances psychological and comprehensive well-being. After the yoga exercise intervention, college students mentioned: “I feel hopeful, strong, and positive”; “I feel that there is always something in life and the world worth loving”; “After yoga class, I feel thoroughly relaxed, energy restored, erasing the fatigue of long hours of study, not tired but more relaxed and full of vitality.” These results indicate that yoga exercise intervention improves physical vitality.

After completing the course, one student described in an interview: “When doing partner yoga, I am willing to support her with all my strength, even if it hurts”; “Helping her with postures beneficial for her health, I feel our relationship has become closer”; “There’s a magical feeling when we leverage each other’s strength because we are giving energy to each other, feeling very warm”; “When her body is fully reliant on me, I am willing to give my utmost effort to help her complete the movement..” However, descriptions of altruistic behavior in meditation diaries were not as vividly presented, indicating that yoga meditation focuses more on individual growth and transformation than the altruistic behavior in partner yoga postures.

## 4 Discussion

Yoga is a holistic exercise that concerns the body and mind, helping balance students’ study, life, body, and mind. Through physical postures, breath control, meditation, and relaxation techniques for psychological adjustment, it achieves harmony and unity of body, mind, and spirit, easing tension and life stress. It has a noticeable improvement effect on negative effects among college students, such as anxiety and depression (Cramer et al., 2013; Domingues, 2018). The results showed that yoga exercise intervention improved the level of nine dimensions of comprehensive well-being among female college students, in which three dimensions of subjective well-being (life satisfaction, positive affect, negative affect) and six dimensions of psychological well-being (health concerns, personality growth, life vitality, altruistic behaviors, friendly relationships, and self-worth) were significantly improved after yoga exercise intervention.

### 4.1 Yoga exercise intervention contributes to enhancing students’ comprehensive well-being

The efficacy of the four specific contents of the yoga exercise intervention includes yoga postures, yoga meditation, breathing, and relaxation techniques. Backbend postures were filled with energy and well-being, expanding the breathing space and aiding in releasing fear, anxiety, and stress (Iyengar, 2008). Twisting poses offered internal stability, helped release emotional tension with a strong balancing effect, stretched the back muscles, and massaged the internal organs (Iyengar, 2008). Partner yoga emphasizes collaborative effort to complete the yoga session in a harmonious and supportive setting, enhancing mutual pleasure, coordinating relationships, fostering friendships, and exercising both body and mind (Iyengar, 2008).

Yoga meditation induces changes in the autonomic nervous system, such as altering heart rate, increasing parasympathetic activity, and reducing sympathetic activity (Uikey and Sandel, 2023). Meditation improves autonomic function by triggering neurohormonal mechanisms, thus reducing stress and anxiety. It also suppressed sympathetic activity by downregulating the hypothalamic–pituitary–adrenal axis (Palek, 2020). Yoga meditation practices induced neural activation in the left prefrontal cortex of participants, stimulating neural activity in the frontal lobe cortex during the process, maintaining an appropriate state of arousal, enhancing neural activity, and thus enhancing neural activation in the left prefrontal cortex of the brain related to cognitive tasks and attention control (Schiweck et al., 2019). Yoga practices result in an excitation-inhibition-excitation process of brain electrical activity, with brain waves displaying an orderly rhythm of synchrony and desynchrony, serving as an effective means to regulate the balance and unity of “body, mind, and spirit” (Brown and Gerbarg, 2005).

Yoga breathing exercises are a unique and powerful way to adjust the imbalanced autonomic nervous system (Uikey and Sandel, 2023). Conscious breathing practices positively affected autonomic nervous functions such as heart rate variability and cardiac vagal tone, influencing emotions, stress, and cognition and improving psychological disorders. Sarika et al. noted that cardiac vagal tone could be a marker for emotional regulation and psychological adaptability (Lemay et al., 2019). Palek et al. suggested that vagal nerve activity is related to attention, emotions, and communication, with vagal effects calming both physiologically and psychologically (Cramer et al., 2017). Schiweck et al. (2019) further confirmed the correlation between depressive moods and reduced cardiac parasympathetic control. Assessing the autoregulatory effects of yoga breathing, the interaction between brain, autonomic, and psychological functions was enhanced, influencing well-being and emotional regulation through the central and parasympathetic nervous systems (Brown and Gerbarg, 2005). Meditation and relaxation processes gently stimulate the central nervous system, keeping the cerebral cortex in an appropriate state of arousal conducive to activating spontaneous electrical activity in brain cortical neurons. The state during yoga meditation is deep quietude and high alertness, with low oxygen consumption, a unique brain functional state. Long-term practice of yoga meditation can enhance neuronal metabolic capacity (Cahn and Polich, 2006).

### 4.2 Yoga exercise intervention can improve the levels of the nine dimensions of comprehensive well-being

Life satisfaction primarily refers to an individual’s satisfaction with their state of life, where their various needs are essentially met. Previous studies have indicated that yoga postures mainly improved life satisfaction (Cramer et al., 2017; Lemay et al., 2019). However, our study presented different results. We found that the life satisfaction of subjective well-being in the yoga group showed a highly significant improvement attributed to the role of yoga meditation. Life satisfaction was mainly enhanced through yoga meditation practices, with meditation improving mood and increasing satisfaction with life, enabling individuals to handle any issue in life with ease and be satisfied with the people and events around them, which differs from some previous research findings.

This study also found that yoga interventions could significantly enhance college students’ subjective well-being regarding positive affect, such as happiness, pleasure, and joy, and reduce negative affect, such as anxiety, stress, and irritability, consistent with previous research findings (Harsora and Nanduri, 2022). When yoga and meditation were used as relaxation and stress relief methods in a busy and pressured life, they helped college students enhance positive affect and alleviate anxiety and restlessness. Interviews revealed an improvement in positive affect among college students and relief from negative affect. Yoga practitioners achieve a state of spiritual awakening through meditation practice, entering an unbounded state of consciousness and reaching the most relaxed and pleasant psychological state, effectively easing anxiety, depression, and other negative effects. Yoga interventions significantly enhanced the health concern dimensions of psychological well-being, in line with previous research results. Yoga can increase college students’ attention to health, improve sleep quality, reduce staying late, and encourage healthy eating, thus maintaining good lifestyle habits and behaviors (Crovetti and Rielly, 2017). Thematic yoga interventions make participants’ diets healthier by healing poor life routines, improving both physical and psychological health, thereby effectively enhancing psychological well-being.

Personality growth mainly refers to self-acceptance, continuous development, and control over one’s behavior and emotions (Dasgupta et al., 2023). Previous research has not explored the dimensions of personality growth extensively. This study shows that the personality growth of psychological well-being in the yoga group scored the most significant change in the comprehensive well-being scale, primarily through yoga practice enhancing emotional regulation and stabilizing emotions. The practice of postures and meditation allowed students to accept, reflect on, and correct their bodily actions and thoughts. Yoga meditation effectively helped participants feel their emotions and learn to accept them, experiencing changes in their mental state during meditation, gradually turning yoga meditation into a habit, fostering personality growth, and enhancing psychological well-being. Self-worth refers to believing in one’s abilities, feeling valued, and having high self-esteem (Lawrence and Gonzales, 2023). The improvement of self-worth through yoga is primarily reflected in bodily self-esteem (Golec de Zavala et al., 2017). Practicing yoga postures increases confidence, and through persistent effort, achieving postures that were previously unattainable and breaking personal limits brings great satisfaction and a sense of accomplishment.

Friendly relationships mainly refer to amicable and harmonious interpersonal relationships (Phu, 2019). After practicing yoga, friendly conversations increase among participants, enhancing social adaptability and the ability to manage interpersonal relationships (Kaur et al., 2021). In this study’s yoga intervention, the dimension of friendly relationship scored significantly higher. Interviews revealed that through the cooperative interaction of partner yoga, completing a yoga session together fosters elements of mutual aid, harmony, and trust that solo yoga does not provide. Current research does not directly link yoga meditation to friendly relationships. However, this study found through meditation diaries that yoga meditation fosters friendly relations among practitioners.

Life vitality primarily refers to being full of energy, enthusiasm for life, and brimming with energy, a state that vibrant college students should inherently possess (Das et al., 2020). Previous research on yoga enhancing life vitality is scarce, with a few studies suggesting that backbend postures primarily improve physical vitality (El Refaye, 2022). In this study, altruistic behavior primarily refers to the willingness to help others. Since yoga postures and meditation practices are usually performed individually on a mat, quietly focusing on one’s practice, previous research has seldom discussed enhancing altruistic behavior through yoga. This study made appropriate adjustments to the practice form of yoga by combining Hatha yoga with partner yoga, changing the individual practice in postures and meditation to designing partner yoga postures. This design allowed participants to learn to perform small acts of altruism, feeling warmth and well-being through mutual support and protection in partner yoga, enhancing communication, physical touch, and concerted efforts to complete the partner yoga postures, helping individuals gradually form a stable pattern of altruistic behavior. It was observed that there was a significant improvement in the altruistic behavior and psychological well-being of the participants.

## 5 Conclusions and recommendations

### 5.1 Conclusion

Yoga exercise interventions can be divided into four parts: postures, meditation, breathing, and relaxation techniques. Through yoga exercise interventions, among the nine dimensions of comprehensive well-being, female college students showed highly significant improvements in subjective well-being (including life satisfaction, positive affect, and negative affect) and two dimensions of psychological well-being (health concern and personality growth), with significant improvements in the other four dimensions of psychological well-being (life vitality, altruistic behavior, friendly relationships, and self-worth). Yoga exercise interventions help enhance the comprehensive well-being of female college students. In addition, yoga meditation focuses on the individual’s growth and transformation, with the improvement in life satisfaction of female college students’ subjective well-being mainly achieved through yoga meditation. Finally, partner yoga posture practice can help individuals gradually form a stable pattern of altruism and be willing to help others. Yoga exercise interventions can be an effective guidance method for college students’ mental health education in universities.

### 5.2 Recommendations

Based on our findings, we make the following recommendations. Firstly, universities should highly value the positive effects of physical exercise interventions on enhancing students’ comprehensive well-being, promote the deep integration of physical and health education, strengthen guidance and supervision over students’ extracurricular physical activities, and push for the continuous development and improvement of college student’s mental health levels. Additionally, further refine the methods, means, and strategies of school mental health education, incorporating yoga and other physical exercise interventions into the mental health education of college students. It’s necessary to provide appropriate education and guidance according to different genders, actively carry out exercise intervention work for the comprehensive well-being of college students, and help students achieve higher well-being. Finally, future research must strengthen the development of localized measurement tools for well-being, focus on more specific sports projects and forms of physical exercise, not only examining physiological and psychological factors but also considering the social factors of physical exercise in enhancing college students’ sense of well-being, the differences in the effects of different dimensions of sports participation on different dimensions of well-being, the differences in the impact of physical exercise on well-being between genders, the mediating effect of peer relationships, and other research topics, to provide decision-making references for the construction and implementation of the “Healthy China” strategy.

## 6 Research limitations

Several limitations should be noted. First, this study was limited by the research methods: In this study, the control group did not engage in any structured physical exercise program during the intervention period to highlight the specific impact of yoga on overall well-being. The absence of a comparison group engaging in other forms of physical exercise may limit the specificity of the conclusions regarding the impact of yoga on the overall well-being of female college students. Future research could include a comparative exercise group and enhance the analysis of gender differences between male and female college students. By distinguishing the effects of yoga from other physical activities on the overall well-being of college students, a more comprehensive and systematic understanding of yoga’s impact on the overall well-being of male and female college students can be achieved. Second, this study was also limited by the sample size and duration of yoga intervention: The subjects of this study were 92 female college students from a university in Shanghai, which is a relatively small sample size. Additionally, the 10-week intervention period might need further validation to determine if it should be extended. Future studies could consider increasing the sample size and number and choosing a longer follow-up period. This design would enhance the external validity of the research results and further assess the time effects and lasting impacts of yoga practice.

## Data availability statement

The original contributions presented in the study are included in the article/supplementary material, further inquiries can be directed to the corresponding author.

## Ethics statement

The studies involving humans were approved by Shanghai Normal University Approval document of academic ethics and ethics Committee. The studies were conducted in accordance with the local legislation and institutional requirements. The participants provided their written informed consent to participate in this study.

## Author contributions

LL: Formal analysis, Resources, Writing – original draft, Writing – review & editing. DL: Data curation, Investigation, Resources, Validation, Writing – review & editing. CL: Conceptualization, Data curation, Funding acquisition, Project administration, Resources, Supervision, Visualization, Writing – original draft. YS: Data curation, Formal analysis, Investigation, Methodology, Supervision, Writing – review & editing.
